# Economic Profits Enhance Trust, Perceived Integrity and Memory of Fairness in Interpersonal Judgment

**DOI:** 10.1371/journal.pone.0051484

**Published:** 2012-12-12

**Authors:** Keisuke Eto, Shigeru Watanabe, Hideaki Kawabata

**Affiliations:** Department of Psychology, Keio University, Mita, Minato-ku, Tokyo, Japan; French National Centre for Scientific Research, France

## Abstract

Does money lead to trust in personality and intention of others? Humans have a strong tendency to judge the intention of others from their sequent behaviors. In general, people trust others who behave fairly, but not always. Here we show that judgments of both intentional aspects and memory of intentional behavior are automatically influenced by unintentional benefits from the behaviors of others. We conducted a reward-manipulated and repeated trust game by using real participants interacting with moving image partners on a computer screen. The participants assessed likability, trustworthiness, and perceived integrity of the partners in pre- and post-game questionnaires. The results of judgments of all three dimensions and the memory of frequency of each partner's fair behavior (sharing) were strongly influenced by profitability in the trust game, even though all partners shared 75% of the profit and participants were told that profitability was randomly assigned to each partner. Furthermore, these effects were moderated by the gender of the participants: males were more sensitive to monetary profits than were females. The results reveal that humans automatically trust, approve the integrity of, and recall well the fair behavior of others who provide affectively positive outcomes such as monetary profits. We call this phenomenon the “affect ripple effect”.

## Introduction

People generally trust and recognize others who behave fairly [1]. Previous studies using investment-game experiments demonstrated that participants form a likable impression and highly trustworthiness of other players, depending on the fair behavior of the participants [2], [3], [4], and they strongly remember whether the other players behaved fairly or unfairly [2], [5]. Moreover, one may infer and judge others' intentions based on their behavior [6]. The ability of inferring others' mental state, such as theory of mind, is a central function in interpersonal perception [7], [8], which may enable us to see behaviors counter to the socially accepted norm [9].

However, in having continuous interactions with others, someone who provides benefit for others may be trusted and recognized to have integrity even if he/she actually does not behave fairly or is not cooperative, such as a selfish but effective and beneficial leader. For example, a useful job ability, more than intentional fairness, is a better predictor of trustworthiness of workers in a workplace [10]. Thus, we need additional explanation for the reason why a person even behaving not fairly is trusted or recognized his/her integrity by someone.

Here we focus on effect and attribution of affect occurred by other's beneficial behavior on social judgment and memory. Because an affective impression influences automatically and quickly [11], interpersonal preference such as good or bad may have a strong impact on interpersonal perception [12], [13], [14] and may expand to a multidimensional judgment of personality, such as the halo effect [15]. Affective and subliminal information sometimes unconsciously or automatically manipulates judgment in not only the judgment of visual materials [16] but also of social targets [17]. Furthermore, humans have a strong tendency to attribute the cause of behavior to others performing the behavior rather than their situation. This tendency is known as correspondence bias [18]. Taking the affect effect into consideration, humans may attribute not only behaviors but also the affective outcome of the behaviors unconsciously. In addition, humans recall selective or even false memory to justify a decision made later [19], [20]. Thus, it is possible that affective interpersonal judgments also influence interpersonal memory, in which a positive affect strengthens positive memory, including behavioral fairness.

There is now common agreement that the trust game, in which two people interact in an economic investment game, is one of the useful methods for researching not only economic behavior but also interpersonal judgments and memory in social relationships [3], [4], [5], [21]. We used a modified version of the trust game to characterize the relationship between interpersonal judgments, memory, and another's profitability as results of the behaviors of others. Regarding investment behaviors in trust game, people are generally supposed to take more risks when they believe that the expected monetary return from the partner is higher, and these beliefs being influenced by their previous monetary experience with this partner. Furthermore, we expected this monetary experience may automatically influence the judgments of intention and memory of the partner by affective effect. So, we hypothesized that the economic profitability of partners influences judgments of intention and memory of the intentional behavior of a partner, even when the perceiver obviously realizes that the profitability is logically not related to the target intention.

In the present study, we conducted a repeated trust game in which participants play 12 times with each 15 partner who were individually presented as a moving image on a computer screen. In the trust game, each partner had been assigned randomly an “Multiplier Rate (MR)” from a set of seven different rates (0 to 12) and a null condition. Investment is automatically multiplied by the MR as an experimental condition, profitability. Immediately before and after the section of the repeated trust game, participants assessed likability, trustworthiness, and perceived integrity of that partner. Therefore, the pre-game partner judgments were elicited before knowing the value of the MR. After the post-game partner judgments, participants answered a questionnaire that included memory tests asking the frequency of the shared behavior, the reward magnitude from each partner, and debriefing items.

## Methods

### Participants

Fifty-two Japanese students living in the Tokyo metropolitan area participated in the experiment (32 females, mean age ± SD: 21.15±1.38 yrs). All of them were tested individually, paid cash for participation (median: 1,350 yen  =  approximately $16, range ±350 yen) based on the result of each trust game. All procedures were accordance with the Declaration of Helsinki, were approved by the local ethical committee of Keio University, Japan. Written consent was obtained from each participants prior to the experiment.

### Stimuli

Fifteen face photographs and the same number of moving images were acquired from a separate group of participants, whom we recruited only for this purpose in Nagoya (in central Japan), which is a different area from Tokyo (in eastern Japan). All 15 persons were Japanese males (mean age ± SD: 23.93±1.29) and were dressed in business shirts. We orally confirmed that no partner was the acquaintance of any participants in the end of each experiment.

### Procedure

The experiment was divided into five parts: instruction, pre-game partner judgments, trust game, post-game partner judgments, and debriefing questionnaire. First, in the instruction phase, participants were asked to answer a 6-item scale (α = .81) for “general trust” [22] with the 7-point Likert-type scale of 1 (disagree strongly) to 7 (agree strongly). A sample item includes “Most people are basically honest”. General trust, as a kind of belief about others, may influence social judgment, as individuals who are high in general trust initially trust others more strongly but judge the other's behavior more strictly than individuals who are low in general trust [22], [23]. We aimed to test whether this belief effect or a moderate MR effect affected social judgments in continuous interactions, such as in a repeated trust game.

Then, they were given instructions (See Appendix S1) on the experimental procedure and the rules of each task, and they were informed that during the trust game they would earn units of experimental currency that would be paid to them in cash at the conclusion of the experiment. Participants were given explanations of the possible range of participation. All tasks except the instructions were presented on a computer via E-Prime 2.0 software (Psychology Software Tools, Inc., Pittsburgh, PA).

### Partner Judgments

**Figure 1:**
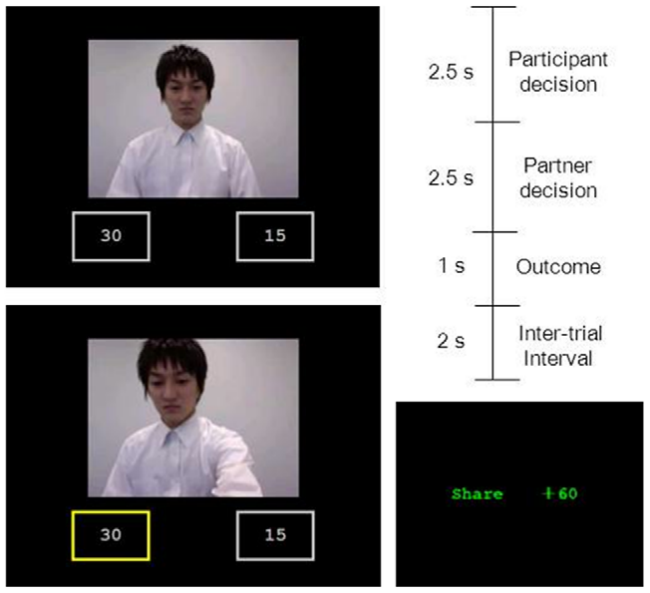
Experimental design of partner judgments. (A) The scale for likability and trustworthiness. (B) The scale for perceived integrity. The parson depicted in the photograph has given written informed consent, as outlined in the PLoS consent form, to publication of his photograph.

In partner judgments pre- and post-game, participants saw photographs of the faces of the partners and assessed likability (*α* = .91), trustworthiness (*α* = .90), and perceived integrity (*α* = .88; see Fig. 1). Perceived integrity was assessed by using a different type of scale from likability and trustworthiness because we tried to test the consistency of our result among different measurements (Table. S8 presents all the items, Cronbach's alpha, and factor loadings of likability and trustworthiness). We measured likability and trustworthiness using four items, including one reverse item, on the 7-point Likert-type scale with anchors of 1 (disagree strongly) to 7 (agree strongly). Some studies suggested that likability and trustworthiness are similar dimensions [14], [24] and actually had strong relationship in this studies, as expected (Pearson's *r*(728) = 0.767, *p*<.001; df (728) was result from the data quantity in which all partners' judgments except null condition (n = 14) of all participants (n = 52)), while some studies treated trustworthiness as a different concept from simple likability or preference [10], [25]. To assess the discriminant validity of these in this study, we first conducted a factor analysis with judgment data using likelihood method factoring and promax rotation. Consistent with above, the first factor accounted majority of the variance (factor 1 = 58.31%; factor 2 = 4.41%) while the factor loadings of items were clearly separated. The 2-factor solution accounted for 62.72% of the variance in the items and the overall fit statistics of this model were good: χ^2^ (13) = 25.743, *p* = 0.018. We measured perceived integrity using four items, including one reverse item on a 7-point Likert-type scale with anchors of 1 (extremely low) to 7 (extremely high). The measurements were displayed on a screen and participants assessed partners by pushing a number button on the keyboard. Participants saw 12 item blocks of 15 faces and, therefore, had 180 judgments pre- and post-game. This task had no time limitation. The order of presentation of blocks and also the faces in each block was randomized.

### Trust Game

**Figure 2:**
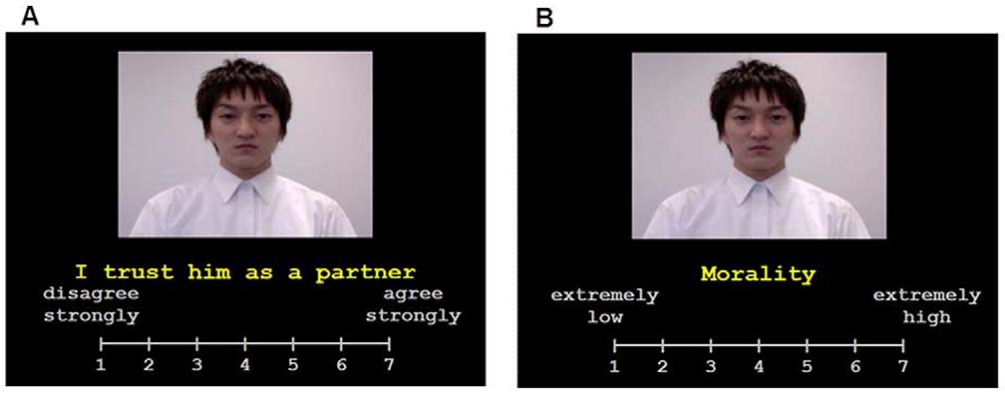
Experimental design of trust game. A trial in the game was divided into a decision phase and an outcome phase and was played with moving image partners. During the decision phase, the chosen option by participants was required within 2.5 s after the start of the trial. The parson depicted in the photograph has given written informed consent, as outlined in the PLoS consent form, to publication of his photograph.

In each game trial, a “participant” played with one “partner”. In each trial in the trust game, the participant plays with one of the partners and first chooses a high risk (30 UEC) or a low risk (15 UEC) investment with the partner (Fig. 2). This investment is automatically multiplied by the “Multiplier Rate (MR)” as an experimental condition. Then the partner can either keep the entire multiplied investment or share the investment with the participant half and half. A key manipulation was that each partner had been assigned randomly an MR from a set of seven different rates (0, 2, 4, 6, 8, 10, and 12) and a null condition, all of which were unknown to the participant at the start of the experiment. Each MR condition was assigned to two partners and the null condition was assigned to one partner. When the participant chose the 30 UEC investment and the partner chose to share, the average outcomes of each MR condition for the participant were as follows: MR0 partner = −30 UEC (because the invested 30 UEC is lost), MR2 = 0 UEC, MR4 = 30 UEC, MR6 = 60 UEC, MR8 = 90 UEC, MR10 = 120 UEC, and MR12 = 150 UEC. The outcome, which fluctuated within a small range of less than 15% (e.g., MR8: 82–98 UEC in each MR except MR1 (always 0 UEC) when the partner shared). When the partner chose keeping, the participant lost the investment in this trial (30 or 15 UEC) regardless of the partner's MR. In the trial with the null condition partner who did not respond and was just displayed in the movie, the UEC of the participant did not change. The frequency of reciprocity, which is an intentional behavior of all partners, was controlled to 75% sharing and 25% keeping, except for the null condition partner. Each participant played 4 blocks of 45 trials for a total of 180 trials. Each trial was divided into a participant's decision phase (2.5 s), a partner's decision phase (2.5 s), and an outcome phase (1 s). During the decision phase, participants viewed moving images of a partner and options (“30” or “15”). During the outcome phase, a behavioral outcome (“share” or “keep”) and a monetary outcome (amount of loss/gain) were presented. During the outcome phase of the null condition partner, “N/A”, which means no answer, was presented.

In the instruction, partners were told that they were playing a trust game with 15 partners as moving images taken in another experiment for this study. Then, explanations were given about the rule and procedure of the trust game. Importantly, participants were told that the MR of each partner was assigned randomly by computers and their responses were the same as the actual behavior recorded in the trust game in the initial experiment in which partner movies were taken. After the explanation, participants had 8 practice trials of the trust game with photographs of different males from partners. We confirmed that all participants understood the rule of the game after the practices.

### Questionnaire

In the last phase of experiments, participants answered the computerized questionnaire, which included two memory tests and debriefing items. In the first memory test, participants chose the share ratio (0% to 100%) of each partner during the trust game. In the second memory test, participants chose the reward magnitude (−60 to +150 UEC) at the time participants had invested 30 UEC and the partner shared. In each test, the order of presentation of the partners was randomized. The debriefing items were four items, including “How do you feel when a high/low MR partner shared/kept during the trust game?” on the 7-point Likert scale (1 =  very bad feeling, 7 = very good feeling) for the manipulation check.

### Manipulation check by debriefing items in the questionnaire

A two-way repeated analysis of variance (ANOVA) revealed a significant interaction (F(1, 51) = 40.34, *p*<0.001) between the MR and the partner response. Simple tests of the main effect revealed that the ratings for high MR partners (6.25) were significantly higher than those for low MR partners (4.94) at the time that the partner shared (F(1, 51) = 112.36, *p*<0.001), while not when the partner kept (high MR: 2.44; low MR: 2.69; F(1, 51) = 1.57, *p* = 0.22). These results suggest our manipulation of profitability could correctly determine affective effect in perceivers more or less in the trust game because the reward magnitude was larger in high MR partners than in low MR partners when partners shared and was the same when partners kept.

### Data Analysis

We tested for normality using the Kolmogorov-Smirnoff test. The variables (likability, trustworthiness and perceived integrity in pre- and post-game, and memory of fair behavior) of average of each faces were normally distributed (p>.20). Because sample size was too large considering data face by face, we could not use Kolmogorov-Smirnoff test, checked visually histograms figure and also confirmed these all variables normally distributed.

ANOVA was conducted to assess the effect of MR as within-subjects factors, and gender as a between-subjects factor on interpersonal judgments, memory, and risk-taking behaviors. The statistical threshold of P<0.05 (two-tailed) was applied for all statistical tests. Furthermore, a series of structure equation models (SEM) was performed to investigate the relationship between independent variables (pre-game judgments, MR, gender) and dependent variables (post-game judgments, memory, risk-taking behaviors). We firstly input profitability which is an inner factor as an mediator in one factor model, then input facial impression in two factor model. Finally, we added gender in two factor model of SEM.

## Results

### Partner Judgments

**Figure 3:**
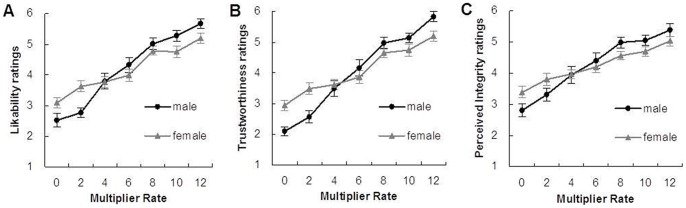
Partner judgments in three dimensions in post-game test on the 7-point Likert-type scale. Graphs show the mean ± SEM. (A) Likability ratings. (B) Trustworthiness ratings. (C) Perceived integrity ratings.

In support of our hypothesis, namely, that profitability has an effect on interpersonal judgments, a two-way ANOVA revealed the main effect of the MR and, interestingly, the moderating effect of gender in post-game partner judgments. When the partner's MR was higher, participants gave a higher post-game likability rating of the partner (F(6, 50) = 55.75, *p*<.001; Fig. 3A). A significant interaction was found between the MR and gender (F(6, 50) = 4.17, *p*<.001): the likability ratings were more strongly related with the MR for male participants than for female participants. Similarly to likability, the analysis revealed a main effect of the MR (F(6, 50) = 66.25, *p*<0.001; Fig. 3B) and a significant interaction (F(6, 50) = 5.35, *p*<0.001) in the post-game judgment of trustworthiness. Furthermore, the MR (F(6, 50) = 32.62, *p*<0.001; Fig. 3C) and a significant interaction (F(6, 50) = 2.43, *p* = 0.026) had a strong effect in the post-game judgment of perceived integrity. The analysis of the pre- and post-game changes of judgments revealed results similar to the post-game judgments. (The results are provided in the Table S1. Participants increased their ratings for higher MR partners, and males were more sensitive to the MR of their partners than were females.

We conducted post-hoc tests (Bonferroni correction) in case a significant main effect was obtained by analysis. Post-hoc tests revealed a strong effect of the difference between the Multiplier Rate (MR) on partner judgments post-game (Table S2). We also conducted post-hoc tests for the change of ratings in partner judgments from pre- to post-game (Table S3).

The analysis of the pre- and post-game changes of judgments revealed results similar to the post-game judgments. A two-way ANOVA revealed the main effect of the MR and also the moderating effect of gender on the change of the partner judgments from pre- to post-game. As the partner's MR was higher, participants changed likability ratings more positively from pre-game to post-game (F(6, 50) = 37.63, *p*<0.001; Fig. S1A), which also had a significant interaction between the MR condition and gender (F(6, 50) = 3.26, *p*<0.01). Similar to likability, the analysis reveals a main effect of MR (F(6, 50) = 42.63, *p*<0.001; Fig. S1B) and a significant interaction (F(6, 50) = 3.23, *p*<0.01) in the change of judgment in trustworthiness. Furthermore, the MR had a main effect (F(6, 50) = 25.38, *p*<0.001; Fig. S1C) but not a significant interaction (F(6, 50) = 1.20, *p* = 0.307) in the change of judgment in perceived integrity. (The result of a simple main effect test is in Table S5).

**Figure 4:**
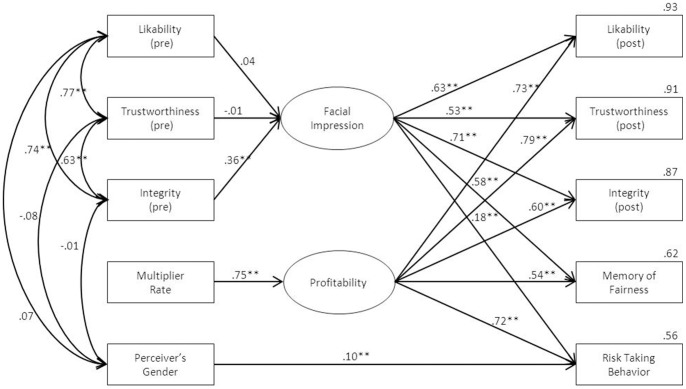
Final model of structure equation models of relationships among MR, profitability and other variables on all partner judgments. * *p*<0.05, ** *p*<0.01.

To analyze the relationships among MR, profitability, and other variables on all partner judgments, we conducted a series of SEM (Table S7). The MR determines the reward magnitude from each partner as his behavioral outcomes so that participants would attribute affective profitability to each partner. Therefore, profitability produced by the MR was considered as a strong factor of partner judgments in the model. This assumption was supported strongly by the final model (Fig. 4). The overall fit statistics of this model were good: χ^2^ (24) = 152.79, *p*<0.01, GFI/AGFI = 0.96/0.90, RMSEA = 0.09, RMSR = 0.06, CFI = 0.98. The results revealed that links between all dependent variables and the MRs are mediated by profitability, which is considered an affective factor. Not surprisingly, facial impression was also found to be another affective factor, as is well known [4], [26], [27]. Consistent with the results of ANOVA, the participant's gender was significantly related to the high-risk investment ratio during the games (β = 0.10, P<0.01).

**Figure 5:**
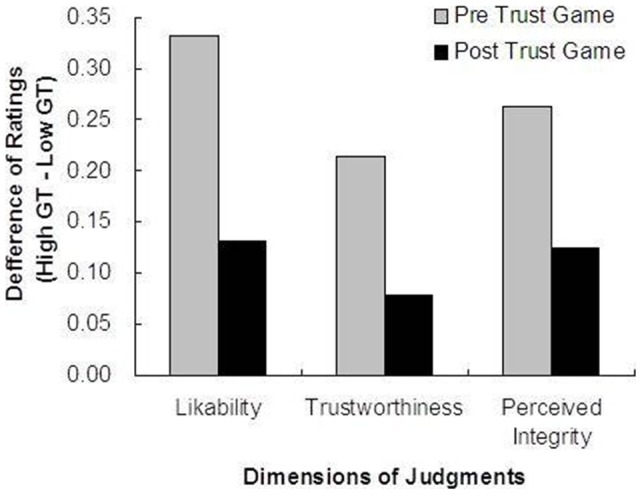
Difference of mean ratings between participants who rated high in general trust (n = 28) and low in general trust (n = 24) in pre- and post-game partner judgments.

To analyze the effect of general trust, we divided participants into two groups: above the mean (n = 28) and below the mean (n = 24) in general trust. As a result of a two-way ANOVA, no significant interaction between MR and general trust was found both in the post-game judgment and the change of judgment from pre- to post-game in all three dimensions. These results suggest the MR effect may be independent from some kinds of conscious beliefs such as general trust. In contrast, we found an expected effect of general trust independent from the MR. Fig. 5 presents the difference of mean ratings between participants who were high in general trust and low in general trust in pre- and post-game partner judgments. The results show that individuals high in general trust were rating highly partners in pre-game likability (t(50) *p* = 0.003), trustworthiness (t(50) *p* = 0.190), and perceived integrity (t(50) *p* = 0.004), but this tendency disappeared in the post-game judgment of likability (t(50) *p* = 0.216), trustworthiness (t(50) *p* = 0.346), and perceived integrity (t(50) *p* = 0.267). This result is consistent with the concept of general trust [22], [23]. We found that individuals high in general trust initially trusted others highly but checked other unfair behaviors severely also in continuous interactions.

In addition, we checked influence of previous experience of interaction with each partner in trust game on post-game partner judgments. The results of analyses did not find significant effect of each partner's last decisions to share or keep on post-game judgment of likability (t(726) *p* = 0.275), trustworthiness (t(726) *p* = 0.852), and perceived integrity (t(726) *p* = 0.310).

### Memory Tests

**Figure 6:**
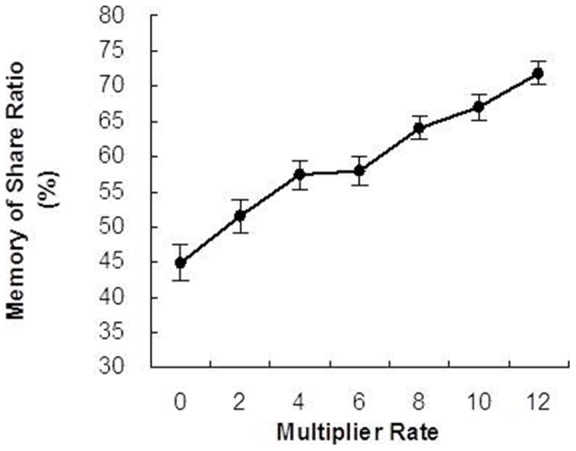
Results of memory tests for the share ratio during the trust game. Graphs show the mean ± SEM. Participants used an 11-point scale (0% to 100%).

Importantly, participants recalled the highest share ratio for the highest rewarding partner, with a decreasing share ratio as the MR decreased. (F(6, 51) = 56.86, *p*<0.001; repeated measures ANOVA; Fig. 6). As expected, we also found a main effect on the memory depending on the reward magnitude of each partner (F(6, 51) = 56.86, *p*<0.001; The results of the post-hoc tests for memory tests are also provided in Table S4).

Interestingly, overall memory of partner's share ratio was lower than actual experience of partners' share ratio (75%) as not expected. The result of memory tests shows a significant difference between actual experience and memory of share ratio even though in high MR condition: MR 12 (t(51) *p* = 0.61) and MR 10 to 0 (t(51) *p*<0.001).

### Investment Behaviors

**Figure 7:**
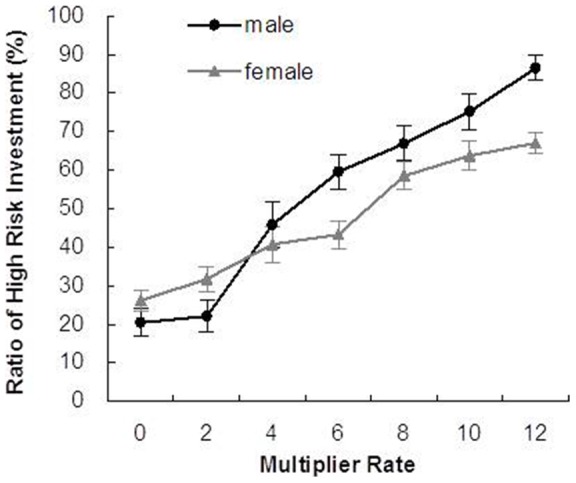
Percentages of high-risk investment (30 UEC) of male and female participants over all trust games. Graphs show the mean ± SEM.

Not surprisingly, the monetary experience with each partner was related to risk-taking behavior of participants. The MR (F(6, 50) = 67.02, *p*<0.001; two-way ANOVA; Fig. 7) had a main effect on the ratio of high-risk investment choice during the games. Post-hoc tests revealed that significant differences of the choice ratio among all MR conditions except for one-level differences. A significant interaction was found as MR effect was stronger in male participants than in female participants (F(6, 50) = 4.64, *p*<0.001; The result of a simple main effect test is in Table S6). A main effect of gender was also revealed as males took high-risk choices more frequently than did females (F(1. 50) = 4.13, *p*<0.05). All male and female participants were students of the Japanese university. No significant difference of age between males (Mean  = 21.4, SD  = 1.465) and females (Mean  = 21, SD  = 1.344) were found (t (50) = 1.009, *p* = 0.318).

Regarding MR of 0, 24% of participant's choice was high-risky investment in spite that they lost the sending money. To check the participants' understandings of the game rules, we compare the ratio of high-risky investment between the first quarter of games (40%) and the last quarter of games (16%) in case of MR 0, which suggests participants took time to remember or learn the profitability of each 15 partners' face more or less rather than they did not understand the rule of trust game. In case of MR 12, participants also largely changed tends of responses from the first quarter of games (58%) to the last quarter of games (85%). Even though some participants cannot remember all 15 sets of each face and MR during limited games, it is obvious that they learn difference of profitability of partners as a whole.

## Discussion

This study demonstrated that humans can trust others who do not behave fairly. The economic profit and loss strongly affected the judgments of likability, trustworthiness, integrity, and memory of fair behavior of the partners, even though the actual sharing ratio (75%) was the same among all partners. Importantly, the result of the memory of fair behavior suggests that perceivers could believe that partners providing more profits have a more cooperative intention in comparison to those providing fewer profits. In other words, humans may really trust the intention of beneficial others. The findings of this study are also supported by industrial research [10] and perceptual study [14].

This effect on interpersonal judgment was moderated by gender: males were more sensitive to monetary profits than were females, which is consistent with other reports of investment games [28]. However, it was slightly modified by “general trust”, which is a kind of conscientious belief. Therefore, this effect from beneficial behavior is independent from some cognitive beliefs and is a biologically fundamental and automatic mechanism.

We found that participants tend to remember more the MR associated to a subject than his previous decisions. Although the MR of each partner was unknown to the participants at the beginning of the trust games, the result of the SEM indicated that the participants attributed their economic outcome to each partner's behavior during the interaction, which may be an affective and inner factor. Furthermore, this affective factor influenced all dependent variables, even though the variables, such as trustworthiness, perceived integrity, and fairness of memory, were logically unrelated to the economic profitability during the games. These effects were consistent with other studies of affective effect [11], [15], [16], [17].

As an extension of the well-known phenomenon of the economic ripple effect, we call this phenomenon the “affect ripple effect” because the affect related to a beneficial outcome has wide effects beyond the logical or meaningful boundaries of various dimensions of social cognition. This phenomenon is not simply explained as an attribution error, such as correspondence bias. In correspondence bias, humans unconsciously have a tendency to attribute behaviors to the actor rather than the situation surrounding the actor [18]. This study demonstrated that humans unconsciously use the outcome of behaviors as reasons of judgments of the actor's trait and the memory of intentional behavior in some conditions. As we manipulated the reward magnitude, the affect underlies this effect. While subliminal priming effect studies [16], [17] strongly support our results, those studies also suggest the effect disappears or contrasts in the case that the priming was conscious to the perceiver and essentially unrelated to the evaluation of the stimuli [29], [30], [31]. Here, we found a strong effect, even though the difference of the reward magnitude was conscious and essentially unrelated to the partner's behavior. Because humans try instinctively to infer other's intentions [6], [7], [8], especially in interactive situations, the inference and memory might be strongly influenced by the affective outcome from other behaviors.

It is noted that we found a negative bias on social memory by the economic outcome, in accordance with prospect theory [32]. Participants recalled keeping behaviors providing monetary loss more often than the actual frequency, even for the highest MR partners. This result suggested that participants had a stronger impression from a loss than from a gain so that they recalled negative behavior more. However, additional studies are needed because the present study could not explore the variations of magnitude, especially of loss.

In addition, we presented a paradigm for studying the relationship among social judgment, memory, decision, and affective outcome or beneficial trait in continuous and interactive relationships, providing that the MR-manipulated trust game and the memory test of the fair behavior ratio are useful methods. For example, although this study emphasizes the affective effect on inference and memory, cognitive control also can influence the affective effect [33]. In this study, such cognitive effort was not counted. Moreover, we used only males as partners of the trust game. Therefore, it is needed to conduct experiment with female partners in order to understand the gender effect more correctly. Finally, it is also possible that some cognitive knowledge or attitude other than General Trust could be used to explore interpersonal judgments and memory of other behaviors. Further explorations in this area would be meaningful.

In conclusion, this study provided crucial evidence of the central reason why the same sequent behaviors of the same person are judged differently by individual people. This implication can also be expanded to the practical effects and reasons of situations, such as salesmen sometimes get trusted by customer entertaining them at great expense even though this is not logically related to their service quality or product, and subordinates are often recognized by their bosses flattering them even though it is an obvious compliment. Moreover, findings in this study shed a new light on and provide additional evidence of the human mechanism, in which interpersonal judgment and memory must be evolutionally based on a fundamental learning mechanism. Interestingly, as with chimpanzees who prefer others who give foods [34], humans automatically not only prefer, but also trust, approve the integrity of, and strengthen the positive memory of others providing affectively positive outcomes to themselves.

## Supporting Information

Figure S1 **Change of partner judgments in three dimensions from pre- to post-game using the 7-point Likert-type scale.** The columns show the mean ± SEM. (A) Change of likability ratings. (B) Change of trustworthiness ratings. (C) Change of perceived integrity ratings.
(TIF)

Appendix S1 **Instructions for Trust Game.**
(DOC)

Table S1 **Means and standard deviations of ratings in partner judgments and memory tests.**
(PDF)

Table S2 **The result of post-hoc tests for main effects of MR on partner judgments post-game.**
(PDF)

Table S3 **The result of post-hoc tests for main effects of MR on the change of ratings in partner judgments from pre- to post-game.**
(PDF)

Table S4 **The results of the post-hoc tests for main effects of MR on memory tests.**
(PDF)

Table S5 **Because of significant interactions between the MR and gender, we conducted a simple main effect test in post-game partner judgments.** Table S5 presents the difference of mean ratings between male and female participants in partner judgments and trust games, and results of the simple main effect tests of gender. In post-game judgments, it is revealed that males made significantly lower ratings only in low MR partners while they made significantly higher ratings only in high MR partners than did females. We conducted simple main effect tests for gender difference in the change of partner judgments from pre- to post-game except for perceived integrity, which did not reveal a significant interaction.
(PDF)

Table S6 **As is consistent with the gender difference in the pre-game partner judgment, a simple main effect test revealed that male participants invested more to a high MR partner and less to a low MR partner than did female participants.**
(PDF)

Table S7 **Summary of fit statistics by a series of SEM.**
(PDF)

Table S8 **Items in Partner judgments.**
(PDF)
