# Supplementary figures and images for: Economic Profits Enhance Trust, Perceived Integrity and Memory of Fairness in Interpersonal Judgment
Source: PLoS One. 2012 Dec 12;7(12):e51484. doi: 10.1371/journal.pone.0051484 (PMC3520791; doi:10.1371/journal.pone.0051484)

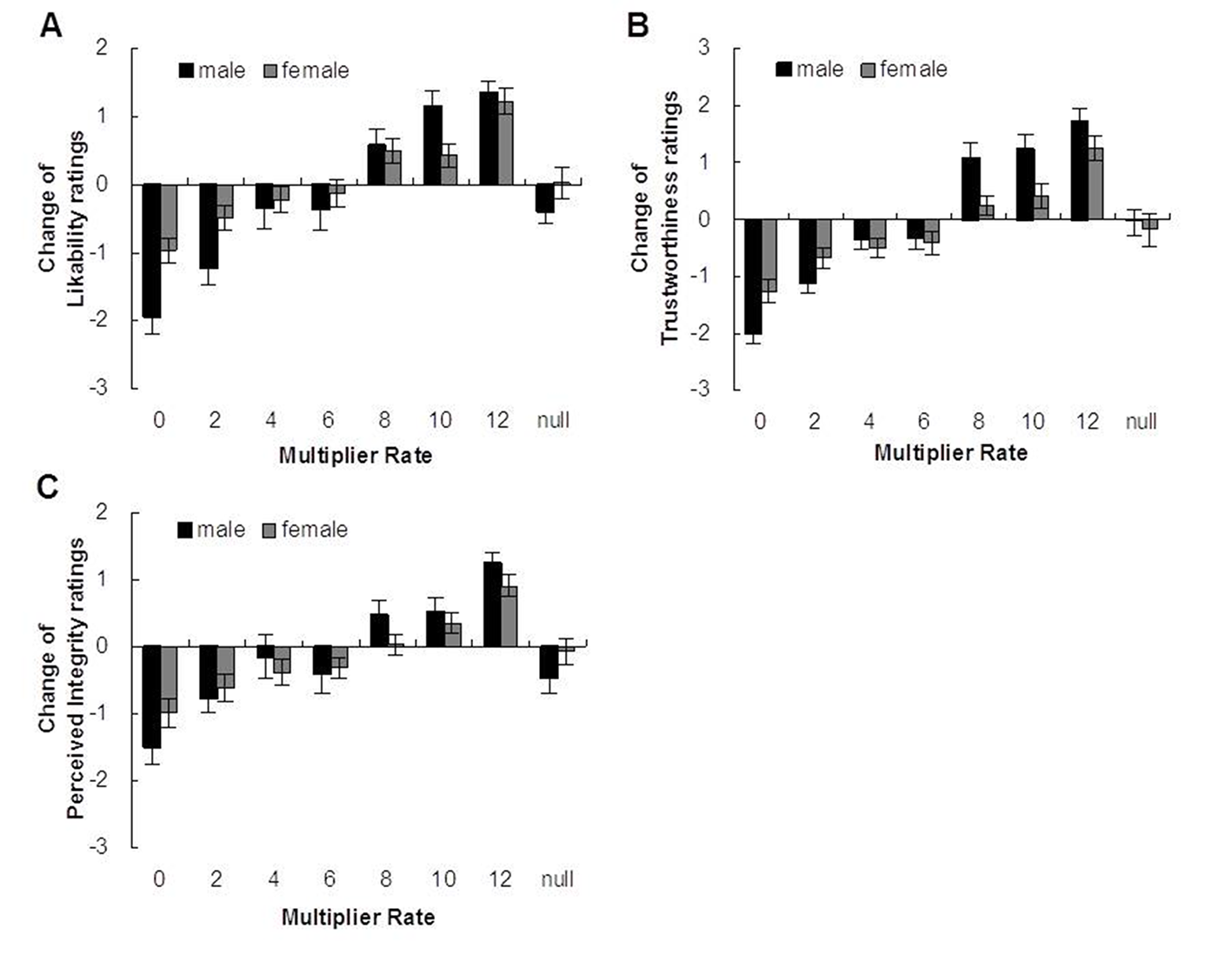

Supplement: Figure S1 — Change of partner judgments in three dimensions from pre- to post-game using the 7-point Likert-type scale. The columns show the mean ± SEM. (A) Change of likability ratings. (B) Change of trustworthiness ratings. (C) Change of perceived integrity ratings. (TIF) [file pone.0051484.s001.tif]
