# Supplementary material for: Economic Profits Enhance Trust, Perceived Integrity and Memory of Fairness in Interpersonal Judgment
Source: PLoS One. 2012 Dec 12;7(12):e51484. doi: 10.1371/journal.pone.0051484 (PMC3520791; doi:10.1371/journal.pone.0051484)
Supplement: Appendix S1 — Instructions for Trust Game. (DOC) [file pone.0051484.s002.doc]

**Appendix S1. Instructions for Trust Game**

In this study, you will play a trust game, which is a kind of computerized investment game with 15 partners who are moving images on the screen. You will have the opportunity to earn money based on your result in the game, so please try to accomplish the highest result possible. The average payment is 1,350 yen and payment ranges from 1,000 yen to 1,700 yen.

**Partners**

All 15 partners are Japanese males (age: 22 to 27 years old) recruited for an initial experiment. They have already played the trust game and movies of them were taken in the initial experiment.

**The rule of the trust game**

You are assigned as an investor and a partner is assigned as a trustee during all trials in this game. The unit of experimental currency (UEC) is used for investments. In each trial, you play the game with a partner one on one. First, the investor makes a choice whether to invest 30 UEC or 15 UEC with the trustee. After the investor makes a decision, the amount of investment is transferred to the trustee. Then, the investment is automatically multiplied by the Multiplier Rate (MR) that each partner has been assigned. The partner can choose either to “keep” the entire multiplied investment or to “share” the investment with you, the participant, half and half. If the partner chooses to keep, you lose the amount of investment you chose (30 or 15 UEC). If the partner chooses to share, you gain the amount deducted your first investment from half of multiplied investment.

Sample Case of Partner’s Multiple Rate = 6

If the investor chose 30 UEC and the partner chose to share, you would gain 60 UEC

If the investor chose 30 UEC and the partner chose to keep, you would lose 30 UEC

If the investor chose 15 UEC and the partner chose to share, you would gain 30 UEC

If the investor chose 15 UEC and the partner chose to keep, you would lose 15 UEC

**Partner’s decision**

Each partner’s decision has already been decided and inputted into the computer by using the actual records of his responses in a trust game in the initial experiment. However, we could not record the responses of one partner because the system was down while taking the moving images. Therefore, one partner will not respond to anything in the game and you will have no outcome in that trial. You just need to choose the 30 or the 15 UEC investment without thinking every time about if the partner is the non-responder.

**Partner’s Multiple Rate (MR)**

Each partner has been assigned an MR randomly from a set of seven different rates (0, 1, 4, 6, 8, 10, and 12) by the computer except for the non-responder. For example, when the participant chooses the 30 UEC investment and the partner chooses sharing, the average outcomes of each MR condition for the participant are as follows: MR0 partner = -30 UEC, MR1 = 0 UEC, MR4 = 30 UEC, MR6 = 60 UEC, MR8 = 90 UEC, MR10 = 120 UEC, and MR12 = 150 UEC. The outcome fluctuates within a small range (e.g., MR8: 82–98 UEC) in each MR condition except MR1. The MR of each partner is fixed through all trials in your experiment.

**Total games and time**

In this experiment, there are 4 blocks of 45 “trials”. It takes approximately 6 minutes for a block and you have a 1-minute break between blocks. Therefore, it takes 30 minutes for a whole trust game. In a block, the partners appear in random order.

**Procedure**

In each trial, you see a decision phase and an outcome phase. You need to push key “1” for the 30 UEC investment and key “2” for the 15 UEC investment during the decision phase. After you make a choice, the partner also pushes a key button on the keyboard**.** Then, the outcomes, which are the partner’s response and the amount you gained or lost, are shown for 1 s. Please be carful that you push a key button within 2.5 s from the start of the decision phase. Otherwise, the 30 UEC investment will be chosen automatically.

Trial Time-Line

Let’s go to practice!!
