# Supplementary material for: Economic Profits Enhance Trust, Perceived Integrity and Memory of Fairness in Interpersonal Judgment
Source: PLoS One. 2012 Dec 12;7(12):e51484. doi: 10.1371/journal.pone.0051484 (PMC3520791; doi:10.1371/journal.pone.0051484)
Supplement: Table S1 — Means and standard deviations of ratings in partner judgments and memory tests. (PDF) [file pone.0051484.s003.pdf]

Table S1. Means and standard deviations of ratings in partner judgments and memory tests

MR	Pre-game partner judgments						Post-game partner judgments						Change of ratings in partner judgments between pre- and post-game						memory tests			
	Likability		Trust-worthiness		Trait integrity		Likability		Trust-worthiness		Trait integrity		Likability		Trust-worthiness		Trait integrity		Share ratio		Reward magnitude	
	M	*SD*	*M*	*SD*	*M*	*SD*	*M*	*SD*	*M*	*SD*	*M*	*SD*	*M*	*SD*	*M*	*SD*	*M*	*SD*	*M*	*SD*	*M*	*SD*
null	3.94	1.29	3.74	1.16	4.15	1.22	3.81	1.01	3.63	1.06	3.93	0.85	-0.13	1.20	-0.12	1.35	-0.23	1.07	42.50	1.89	3.60	1.18
0	4.21	0.94	4.16	0.95	4.36	0.93	2.87	1.03	2.62	0.93	3.16	1.03	-1.34	1.21	-1.54	1.19	-1.19	1.19	44.90	1.86	3.51	1.26
2	4.07	1.07	3.98	0.99	4.29	1.00	3.30	1.01	3.13	1.08	3.61	1.04	-0.77	1.13	-0.84	1.09	-0.68	1.06	51.54	1.69	3.64	0.93
4	4.05	0.82	4.01	0.80	4.26	0.92	3.78	1.04	3.57	1.03	3.96	1.03	-0.27	1.21	-0.44	1.20	-0.30	1.27	57.40	1.48	4.63	1.16
6	4.34	0.75	4.36	0.84	4.63	0.70	4.12	1.16	3.98	1.20	4.27	1.04	-0.22	1.24	-0.38	1.31	-0.35	1.07	57.98	1.45	4.92	1.04
8	4.35	0.87	4.21	0.90	4.53	0.74	4.88	0.82	4.78	0.90	4.72	0.80	0.52	1.03	0.57	1.10	0.19	0.93	64.04	1.14	5.55	0.89
10	4.25	0.91	4.17	1.04	4.42	0.81	4.96	0.97	4.90	1.01	4.83	0.84	0.71	1.06	0.73	1.27	0.41	0.88	67.02	1.31	6.15	1.40
12	4.10	0.88	4.01	0.92	4.14	0.87	5.38	0.88	5.44	0.91	5.17	0.89	1.28	0.95	1.43	1.18	1.03	0.86	71.83	1.18	6.87	1.14

M = Mean; *SD* = Standard deviation
