# Supplementary material for: Economic Profits Enhance Trust, Perceived Integrity and Memory of Fairness in Interpersonal Judgment
Source: PLoS One. 2012 Dec 12;7(12):e51484. doi: 10.1371/journal.pone.0051484 (PMC3520791; doi:10.1371/journal.pone.0051484)
Supplement: Table S2 — The result of post-hoc tests for main effects of MR on partner judgments post-game. (PDF) [file pone.0051484.s004.pdf]

Table S2. Absolute value of difference of mean ratings in post-game partner judgments

MR	0	1		4		6		8		10		12	
Likability ratings in post-game													
0		0.43		0.91	***	1.25	***	2.00	***	2.09	***	2.51	***
2				0.48		0.82	**	1.58	***	1.66	***	2.08	***
4						0.34		1.10	***	1.18	***	1.60	***
6								0.75	*	0.84	**	1.26	***
8										0.08		0.50	*
10												0.42	
12													
Trustworthiness ratings in post-game													
0		0.52	*	0.95	***	1.36	***	2.16	***	2.28	***	2.83	***
2				0.44		0.84	**	1.65	***	1.76	***	2.31	***
4						0.41		1.21	***	1.33	***	1.88	***
6								0.80	**	0.92	**	1.47	***
8										0.12		0.66	**
10												0.55	
12													
Perceived integrity ratings in post-game													
0		0.45		0.80	*	1.11	***	1.56	***	1.67	***	2.01	***
2				0.35		0.66	*	1.11	***	1.22	***	1.56	***
4						0.31		0.76	***	0.87	***	1.21	***
6								0.45		0.56		0.90	**
8										0.11		0.45	
10												0.34	
12													

Asterisks indicate significant difference in a result of post-hoc tests (Bonferroni correction).

* $P < 0.05$, ** $P < 0.01$, *** $P < 0.001$
