# Supplementary material for: Economic Profits Enhance Trust, Perceived Integrity and Memory of Fairness in Interpersonal Judgment
Source: PLoS One. 2012 Dec 12;7(12):e51484. doi: 10.1371/journal.pone.0051484 (PMC3520791; doi:10.1371/journal.pone.0051484)
Supplement: Table S3 — The result of post-hoc tests for main effects of MR on the change of ratings in partner judgments from pre- to post-game. (PDF) [file pone.0051484.s005.pdf]

Table S3. Absolute value of difference of mean change of ratings between pre- and post-game partner judgments

MR	0	1	4	6	8	10	12
Change of likability ratings from pre- to post-game							
0		0.57 **	1.07 **	1.12 ***	1.87 ***	2.05 ***	2.62 ***
2			0.50	0.55	1.29 ***	1.48 ***	2.05 ***
4				0.05	0.79 **	0.98 **	1.55 ***
6					0.74 *	0.93 **	1.50 ***
8						0.19	0.75 *
10							0.57
12							
Change of trustworthiness ratings from pre- to post-game							
0		0.70 **	1.10 **	1.16 ***	2.11 ***	2.27 ***	2.98 ***
2			0.40	0.46	1.41 ***	1.57 ***	2.28 ***
4				0.06	1.01 ***	1.17 ***	1.87 ***
6					0.95 **	1.11 **	1.81 ***
8						0.16	0.87 **
10							0.70 *
12							
Change of perceived integrity ratings from pre- to post-game							
0		0.52 *	0.89 *	0.84 *	1.39 ***	1.61 ***	2.23 ***
2			0.38	0.32	0.87 ***	1.09 ***	1.71 ***
4				0.06	0.49	0.71	1.33 ***
6					0.55	0.77 *	1.39 ***
8						0.22	0.84 ***
10							0.62 **
12							

Asterisks indicate significant difference in a result of post-hoc test (Bonferroni correction).

* $P < 0.05$, ** $P < 0.01$, *** $P < 0.001$
