# Supplementary material for: Economic Profits Enhance Trust, Perceived Integrity and Memory of Fairness in Interpersonal Judgment
Source: PLoS One. 2012 Dec 12;7(12):e51484. doi: 10.1371/journal.pone.0051484 (PMC3520791; doi:10.1371/journal.pone.0051484)
Supplement: Table S4 — The results of the post-hoc tests for main effects of MR on memory tests. (PDF) [file pone.0051484.s006.pdf]

Table S4. Absolute value of differences of recalled reward magnitude in memory test

MR	0	1	4	6	8	10	12
Recalled share ratio in memory test							
0		0.13	1.13 ***	1.41 ***	2.04 ***	2.64 ***	3.36 ***
2			0.99 ***	1.28 ***	1.90 ***	2.51 ***	3.22 ***
4				0.29	0.91 ***	1.52 ***	2.23 ***
6					0.63	1.23 ***	1.94 ***
8						0.61 *	1.32 ***
10							0.71 *
12							
Recalled reward magnitude in memory test							
0		0.13	1.13 ***	1.41 ***	2.04 ***	2.64 ***	3.36 ***
2			0.99 ***	1.28 ***	1.90 ***	2.51 ***	3.22 ***
4				0.29	0.91 ***	1.52 ***	2.23 ***
6					0.63	1.23 ***	1.94 ***
8						0.61 *	1.32 ***
10							0.71 *
12							

Asterisks indicate significant difference in a result of post-hoc tests (Bonferroni correction).

* $P < 0.05$, ** $P < 0.01$, *** $P < 0.001$
