# Supplementary material for: Economic Profits Enhance Trust, Perceived Integrity and Memory of Fairness in Interpersonal Judgment
Source: PLoS One. 2012 Dec 12;7(12):e51484. doi: 10.1371/journal.pone.0051484 (PMC3520791; doi:10.1371/journal.pone.0051484)
Supplement: Table S5 — Because of significant interactions between the MR and gender, we conducted a simple main effect test in post-game partner judgments. Table S5 presents the difference of mean ratings between male and female participants in partner judgments and trust games, and results of the simple main effect tests of gender. In post-game judgments, it is revealed that males made significantly lower ratings only in low MR partners while they made significantly higher ratings only in high MR partners than did females. We conducted simple main effect tests for gender difference in the change of partner judgments from pre- to post-game except for perceived integrity, which did not reveal a significant interaction. (PDF) [file pone.0051484.s007.pdf]

Table S5. Gender difference in partner judgments and trust games

MR	mean ratings in post-game partner judgments						mean change of ratings from pre- to post-game partner judgments					
	Likability		Trust-worthiness		Trait integrity		Likability		Trust-worthiness		Trait integrity	
	M		*M*		*M*		*M*		*M*		*M*	
0	-0.57	†	-0.85	**	-0.58	*	-0.97	**	-0.72	*	-0.52	
2	-0.86	**	-0.92	**	-0.49		-0.74	*	-0.45		-0.14	
4	0.02		-0.12		-0.02		-0.11		0.16		0.24	
6	0.34		0.30		0.19		-0.23		0.09		-0.09	
8	0.22		0.31		0.42	†	0.08		0.84	**	0.45	
10	0.53	†	0.40		0.36		0.73	*	0.83	*	0.17	
12	0.48	†	0.63	*	0.35		0.12		0.45		0.34	

M = Mean. The values are result by male's mean minus female's mean. Asterisks indicate significant difference between genders in results of simple main effect tests.

†P <0.07, *P < 0.05, ** P < 0.01
