# Supplementary material for: Economic Profits Enhance Trust, Perceived Integrity and Memory of Fairness in Interpersonal Judgment
Source: PLoS One. 2012 Dec 12;7(12):e51484. doi: 10.1371/journal.pone.0051484 (PMC3520791; doi:10.1371/journal.pone.0051484)
Supplement: Table S6 — As is consistent with the gender difference in the pre-game partner judgment, a simple main effect test revealed that male participants invested more to a high MR partner and less to a low MR partner than did female participants. (PDF) [file pone.0051484.s008.pdf]

Table S6. Ratio of high-risk investment (30 UEC) of male and female partners over all trust games (%)

Multiplier rate	male	female	difference	
0	20.42	26.17	-5.76	
2	22.08	31.77	-9.69	†
4	45.83	40.76	5.08	
6	59.58	43.23	16.35	**
8	66.88	58.46	8.41	
10	75.21	63.80	11.41	†
12	86.46	67.06	19.40	**

M = Mean. Asterisks indicate significant difference between genders in results of a simple main effect test.

†P <0.07, * P < 0.05, ** P < 0.01
