# Supplementary material for: Economic Profits Enhance Trust, Perceived Integrity and Memory of Fairness in Interpersonal Judgment
Source: PLoS One. 2012 Dec 12;7(12):e51484. doi: 10.1371/journal.pone.0051484 (PMC3520791; doi:10.1371/journal.pone.0051484)
Supplement: Table S7 — Summary of fit statistics by a series of SEM. (PDF) [file pone.0051484.s009.pdf]

Table S7. Summary of fit statistics

Model	χ^2	*df*	χ^2/df	GFI	AGFI	RMSEA	RMSR	CFI
1. One factor model	290.92	24	12.12	0.92	0.84	0.12	0.11	0.95
2. Two factor model	146.87	19	7.73	0.96	0.89	0.10	0.07	0.98
3. Final model	152.79	24	6.37	0.96	0.90	0.09	0.06	0.98

GFI = goodness-of-fit index, AGFI = adjusted goodness-of-fit index; RMSEA = root mean-square error of approximation; RMSR = root-mean-square residual ; CFI = comparative fit index
