# Supplementary material for: Economic Profits Enhance Trust, Perceived Integrity and Memory of Fairness in Interpersonal Judgment
Source: PLoS One. 2012 Dec 12;7(12):e51484. doi: 10.1371/journal.pone.0051484 (PMC3520791; doi:10.1371/journal.pone.0051484)
Supplement: Table S8 — Items in Partner judgments. (PDF) [file pone.0051484.s010.pdf]

Table S8. Items in Partner Judgments

	Items	F1	F2
Likability			
1	He is likable	**.856**	.032
2	He makes a good impression	**.761**	.065
3	I prefer him among the partners	**.749**	.097
4	I do not like him (reverse item)	**.695**	.015
Trustworthiness			
1	I can entrust an important matter to him	-.088	**.854**
2	I trust him as a partner	.197	**.656**
3	I can ask him for help in a pinch	.161	**.637**
4	I cannot rely on him at a group work (reverse item)	.320	**.435**
Perceived integrity			
1	morality		
2	honesty		
3	sincereness		
4	vice (reverse item)		

Factor loadings are presented only for likability and trustworthiness because we used a different scale for the trait integrity. Boldface indicates factor loadings greater than 0.40.
